# Editorial: Covid-19 and Diabetes

**DOI:** 10.3389/fendo.2022.933562

**Published:** 2022-06-13

**Authors:** Mohamed Abu-Farha, Susanna Hofmann, Hamad Ali

**Affiliations:** ^1^ Biochemistry and Molecular Biology Department, Dasman Diabetes Institute, Dasman, Kuwait; ^2^ Institute of Diabetes and Regeneration Research, Helmholtz Diabetes Center, Helmholtz Center Munich, Neuherberg, Germany; ^3^ Department of Genetics and Bioinformatics, Dasman Diabetes Institute, Dasman, Kuwait; ^4^ Department of Medical Laboratory Sciences, Faculty of Allied Health Sciences, Health Sciences Center (HSC), Kuwait University, Jabriya, Kuwait

**Keywords:** COVID-19, diabetes, glycemic control, metformin, diabetes treatment

The COVID-19 pandemic caused by the SARS-CoV-2 virus had a global impact on people’s health especially impacting people with comorbidities. It was immediately established that people with Type 1 diabetes (T1D) or Type 2 diabetes (T2D) had a substantially higher risk of COVID-19 related complications. Additionally, there has been concern that people infected with the SARS-CoV-2 virus may also have a higher risk for developing new onset diabetes. Initial data highlighted and suggested a bidirectional relationship between diabetes and COVID-19 infection. This special issue was designed to further explore this relationship between SARS-COV2 and diabetes to address factors affecting blood glucose impact on people infected with SARS-COV-2, new-onset diabetes, and severe metabolic complications of preexisting diabetes including kidney disease as well as cardiovascular diseases.

Diabetes and obesity are reaching extremely high rates across the globe amounting to a global pandemic. Holly et al. discussed the consequences of this pandemic on the people diagnosed with COVID-19. The authors discussed the consequences of the pre-existing chronic inflammatory state in diabetes and obesity that predispose to the cytokine storm. Obesity and diabetes have been associated with impaired immune response, atherothrombotic state and accumulation of AGEs activating RAGE that hampers the clinical response to SARS-CoV-2 infection. Additionally, the authors also discussed the idea that the virus can exploit mechanisms such as endocytosis *via* the endosomal/lysosomal route to enhance its infectivity. They discussed the role of multiple genes such as ACE2, GRP78 and TMPRRS2 in these pathways. These risk and potential treatments were also discussed by Jin and Hu and Corrao et al.

In continuation, Muniangi-Muhitu et al. proposed the idea of bidirectional link between COVID-19 and diabetes. They discussed the potential mechanisms that can lead to increased severity of COVID-19 in people with diabetes. On the other hand, they also discussed the long-term impact of SARS-COV-2 infection on the development of diabetes by affecting insulin production from pancreatic beta cells and insulin action as well as the roles of the immune system (Muniangi-Muhitu et al.). The role of the immune system was discussed in more details in a mini-review by Rahmani-Kukia and Abbasi. In another commentary, Ardestani and Maedler discussed the use of iPS-derived islet cells to study SARS-COV-2 infection and highlighted the need for more liver, heart and pancreas autopsies from COVID-19 to elucidate viral infectivity and its impact on these organs

Multiple articles looked at the impact of COVID-19 on blood glucose. In a meta-analysis, Chen et al. showed that severe COVID-19 was associated with elevated blood glucose. Similarly, Zhang et al. showed that impaired fasting glucose and diabetes at admission were associated with increased COVID-19 severity. Another finding by Liu et al. showed similar impact of diabetes on COVID-19 but unlike other studies did not demonstrate any increased infection rate amongst people with diabetes in 1,880 COVID-19 patients admitted to Leishenshan Hospital, Wuhan, China. In another small study from China involving 52 patients with diabetes, similar findings were presented where the authors additionally recommended vigorous cardiac troponin testing for diabetes patients with COVID-19 and recommended the use of α-glucosidase inhibitors as a potential protectant for diabetes patients with COVID-19(Zhang et al.). Findings of this study should be taken with caution since it was a small retrospective study. Even in people not diagnosed with diabetes, fasting blood glucose trajectories showed strong association with increased death COVID-19 patients by Song et al. Furthermore, in an opinion article, De Francesco et al. suggested that RAGE/RAGE ligands axis might be an additionally critical factor given its chronic elevation in people with diabetes and its role in innate immunity and coagulation homeostasis [12]. The role of immunological and clinical factors were explored by Han et al. showing that the elevation of cytokines as well as the reduced number of CD8+ T cells and NK amongst other immunological factors can contribute high risk of COVID-19 patients with diabetes.

Multiple treatment modalities such as metformin were also presented in this Research Topic. In a systematic review, Zangiabadian et al. showed that metformin can potentially improve clinical outcomes in COVID-19 patients with mild to moderate infections. This data was further corroborated in a retrospective analysis of 25,326 subjects tested for COVID-19 at Birmingham Hospital, USA by Crouse et al. Tee et al. also highlighted that migrant worker suffer from a high rate of undiagnosed pre-diabetes that increases their risk of pneumonia and electrolyte abnormalities from COVID-19. Association between Plasma level of cystatin C and COVID-19 prognosis was investigated highlighting the need for special attention to people with elevated cystatin C levels especially if they have diabetes Yang et al.

Given the importance of physical exercise in the glycemic control for people with diabetes, Marcal et al. suggested that home-based exercise programs should be also recommended during COVID-19 outbreak for diabetes control. Additionally, Maiorino et al. argued for the beneficial impact of Metatherian diet on people with diabetes and COVID-19 infection given its beneficial anti-inflammatory and immunomodulatory properties. It’s important to mention that this was an opinion article without clinical evidence support. A Quasi-experimental observational trial was also conducted by Lin et al. to show that assisting patients with chronic diseases such as diabetes to maintain good self-management behavior may also contribute to reducing the impact of the pandemic.

Taken together, this collection of articles highlighted the need for effective glucose control and monitoring and explored the bidirectional relationship between COVID-19 and diabetes. They also, discussed possible mechanism of action as well as possible drug target to mitigate the impact of this pandemic on global health.

## Author Contributions

MA-F, SH, and HA Co-edited the special issue. MA-F and HA wrote the editorial. All authors contributed to the article and approved the submitted version.

## Conflict of Interest

The authors declare that the research was conducted in the absence of any commercial or financial relationships that could be construed as a potential conflict of interest.

## Publisher’s Note

All claims expressed in this article are solely those of the authors and do not necessarily represent those of their affiliated organizations, or those of the publisher, the editors and the reviewers. Any product that may be evaluated in this article, or claim that may be made by its manufacturer, is not guaranteed or endorsed by the publisher.

